# Immune Status and Hepatic Antioxidant Capacity of Gilthead Seabream *Sparus aurata* Juveniles Fed Yeast and Microalga Derived β-glucans

**DOI:** 10.3390/md19120653

**Published:** 2021-11-23

**Authors:** Bruno Reis, Ana Teresa Gonçalves, Paulo Santos, Manuel Sardinha, Luís E. C. Conceição, Renata Serradeiro, Jaume Pérez-Sánchez, Josep Calduch-Giner, Ulrike Schmid-Staiger, Konstantin Frick, Jorge Dias, Benjamín Costas

**Affiliations:** 1 SPAROS Lda., Área Empresarial de Marim, Lote C, 8700-221 Olhão, Portugal; AnaGoncalves@sparos.pt (A.T.G.); manuelsardinha@sparos.pt (M.S.); LuisConceicao@sparos.pt (L.E.C.C.); jorgedias@sparos.pt (J.D.); 2 Centro Interdisciplinar de Investigação Marinha e Ambiental (CIIMAR), Universidade do Porto, Terminal de Cruzeiros de Leixões, Av. General Norton de Matos s/n, 4450-208 Matosinhos, Portugal; paulo.santos@ciimar.up.pt; 3 Instituto de Ciências Biomédicas Abel Salazar (ICBAS-UP), Universidade do Porto, R. Jorge de Viterbo Ferreira 228, 4050-313 Porto, Portugal; 4 Sorgal S.A., Estrada Nacional 109, Lugar da Pardala, 3880-728 São João de Ovar, Portugal; 5 GreenCoLab—Associação Oceano Verde, Campus de Gambelas, Universidade do Algarve, 8005-139 Faro, Portugal; 6 Riasearch, Rua do Farol, 131, Torrão do Lameiro, 3880-394 Ovar, Portugal; renataserradeiro@riasearch.pt; 7 Nutrigenomics and Fish Growth Endocrinology Group, Institute of Aquaculture Torre de la Sal, IATS-CSIC, 12595 Castellón, Spain; jaime.perez.sanchez@csic.es (J.P.-S.); calduch@iats.csic.es (J.C.-G.); 8 Fraunhofer Institute for Interfacial Engineering and Biotechnology IGB, Innovation Field Algae Biotechnology—Development, Nobelstrasse 12, 70569 Stuttgart, Germany; ulrike.schmid-staiger@igb.fraunhofer.de; 9 Institute of Interfacial Process Engineering and Plasma Technology, University of Stuttgart, Pfaffenwaldring 31, 70569 Stuttgart, Germany; konstantin.frick@igb.fraunhofer.de

**Keywords:** *Saccharomyces cerevisiae*, *Phaeodactylum tricornutum*, *Sparus aurata*, β-glucans, pulse feeding, immune tolerance

## Abstract

This work aimed to evaluate the effects of dietary supplementation with β-glucans extracted from yeast (*Saccharomyces cerevisiae*) and microalga (*Phaeodactylum tricornutum*) on gene expression, oxidative stress biomarkers and plasma immune parameters in gilthead seabream (*Sparus aurata*) juveniles. A practical commercial diet was used as the control (CTRL), and three others based on CTRL were further supplemented with different β-glucan extracts. One was derived from *S. cerevisiae* (diet MG) and two different extracts of 21% and 37% *P. tricornutum*-derived β-glucans (defined as Phaeo21 and Phaeo37), to give a final 0.06% β-glucan dietary concentration. Quadruplicate groups of 95 gilthead seabream (initial body weight: 4.1 ± 0.1 g) were fed to satiation three times a day for 8 weeks in a pulse-feeding regimen, with experimental diets intercalated with the CTRL dietary treatment every 2 weeks. After 8 weeks of feeding, all groups showed equal growth performance and no changes were found in plasma innate immune status. Nonetheless, fish groups fed β-glucans supplemented diets showed an improved anti-oxidant status compared to those fed CTRL at both sampling points (i.e., 2 and 8 weeks). The intestinal gene expression analysis highlighted the immunomodulatory role of Phaeo37 diet after 8 weeks, inducing an immune tolerance effect in gilthead seabream intestine, and a general down-regulation of immune-related gene expression. In conclusion, the results suggest that the dietary pulse administration of a *P. tricornutum* 37% enriched-β-glucans extract might be used as a counter-measure in a context of gut inflammation, due to its immune-tolerant and anti-oxidative effects.

## 1. Introduction

Aquaculture is the fastest growing food-sector related industry, and as practices become more intensive, the risk of disease outbreaks increases accordingly [1,2]. In fact, animal health-related issues are nowadays the major constraint for aquaculture expansion and sustainability [3]. To date, one of the main strategies to cope with disease outbreaks in aquaculture has been the use of antibiotics. Although this issue has been mitigated in recent years with more restrictive legislation and regulations, antibiotics are still routinely used, leading to the emergence of new antibiotic-resistant bacteria [1]. In addition to vaccination, an alternative strategy to the use of antibiotics is the adoption of prophylactic measures through nutrition, such as the incorporation of immunostimulants and prebiotics in feeds to enhance fish disease resistance and general health [2,4,5].

Marine microalgae are a rich source of bioactive compounds [6] that are drawing increasing attention considering their use in different applications including functional feeds [7,8]. *Phaeodactylum tricornutum* is a marine diatom, unicellular brown microalgae rich in several health beneficial compounds such as β-glucans (BGs) [9,10,11]. BGs can be naturally found as cell wall components in bacteria, yeast, fungi, plants, micro- and macro-algae, and due to their promising biological activities, BGs have been extensively studied in vertebrates [2,12,13,14]. These polysaccharides can act as a prebiotic, enhancing the growth of commensal microbiota and by directly stimulating the innate immune system through interaction with specific cell receptors [4]. BGs bioactivity depends on their degree of branching, size and molecular structure [15]. However, those with higher biological activity show a common pattern: a repeating chain of (l-3)-linked β-D-glucopyranosyl units with randomly branched single β-D -glucopyranosyl units attached by l-6 or 1–4 linkages [2,15]. These repeating patterns, a feature shared with bacterial lipopolysaccharides (LPS), can be recognized by the host’s cell pattern recognition receptors (PRR) and are termed pathogen-associated microbial patterns (PAMPs). Upon recognition. they can elicit an inflammatory response and activate the host’s innate immune cells [12].

In mammals, dectin-1 is the best described BGs receptor, considered to be the most important for recognition and signal transduction. It is a C-type lectin receptor (CLR) which is predominantly expressed on cells from both the monocyte/macrophage and neutrophil lineages [12,16]. In a former study, European common carp (*Cyprinius carpio*) macrophages were activated with curdlan, a dectin-1-specific BG ligand in mammals, showing that immune modulatory effects in carp macrophages could be triggered by a member of the CLR family, although different from dectin-1 receptor [17]. In teleosts, the specific receptors involved in the recognition of BGs and consequent downstream signalling remain to be elucidated [14,18,19,20]. In contrast, the beneficial effects of BGs in fish innate immune response are well documented. Most of the studies focusing on fish showed that oral administration of BGs not only benefits innate immune response, such as the increase of phagocytic capacity, oxidative burst, lysozyme and complement activity [21,22,23,24], but also modulates immune gene expression in different organs [25,26,27]. However, the use of BG-rich microalgae cell extracts as feed supplements to modulate both the systemic and local immune response is still poorly explored. Therefore, the present study aimed to evaluate the effects of β-glucans extracted from microalga (*P. tricornutum*) and yeast (*Saccharomyces* *cerevisiae*), when applied as dietary supplements for juveniles of a valuable fish species for European aquaculture such as gilthead seabream (*Sparus aurata*).

## 2. Results

### 2.1. Growth Performance

Growth performance data are presented in Table 1. At the end of the trial (8 weeks), all fish showed similar final whole-body weight, regardless of dietary treatment. All groups showed similar feed conversion ratio (FCR) and relative growth rate (RGR) values (1.2 and 3.8% day^−1^, respectively).

### 2.2. Haematological Profile and Humoral Parameters

Peripheral cell dynamics were analysed at both sampling points (Table 2). The relative percentage of circulating lymphocytes increased in fish-fed Phaeo21 compared to CTRL and Phaeo37 groups after 2 weeks of feeding. In contrast, the same cell type showed decreased percentages in fish fed MG and Phaeo21 dietary treatments compared to those fed CTRL in the second (8 weeks) sampling. Furthermore, after 8 weeks, peripheral thrombocytes were higher in MG and Phaeo37 compared to CTRL. Monocytes and neutrophils numbers remained unaltered among dietary groups at both sampling points. Plasma humoral parameters (i.e., bactericidal activity, antiprotease activity and IgM) remained unchanged among dietary treatments at both 2 and 8 weeks (Table 3).

### 2.3. Oxidative Stress Biomarkers

**Figure 1:**
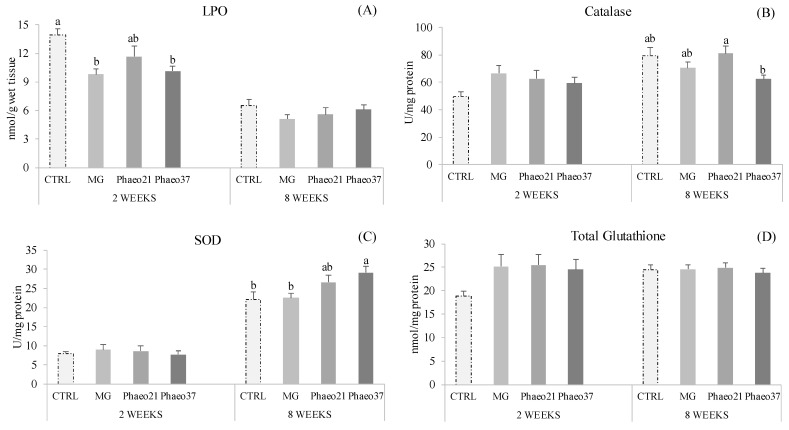
Liver oxidative stress biomarkers of gilthead seabream juveniles after 2 and 8 weeks feeding. Lipid peroxidation (LPO) (**A**); Catalase activity (CAT) (**B**); Superoxide dismutase activity (SOD) (**C**) and Total Glutathione (**D**). Data are the mean ± SEM (*n* = 12). Different lowercase letters indicate significant differences between dietary treatments (*p* < 0.05).

Hepatic oxidative stress biomarkers showed significant differences at both sampling points (Figure 1). Lipid peroxidation decreased in seabream-fed MG and Phaeo37 diets compared to those fed CTRL at 2 weeks (early sampling), while the extent of lipid peroxidation was similar among groups after 8 weeks (final sampling). Catalase (CAT) and superoxide dismutase (SOD) activities were not affected by the dietary treatments at 2 weeks, however, CAT activity increased in Phaeo21 compared to Phaeo37 group at 8 weeks. SOD activity was enhanced in Phaeo37-fed fish compared to those fed CTRL and MG1000 dietary treatments. Total glutathione remained unaltered among dietary groups at both sampling points.

### 2.4. Multivariate Analysis from Physiological Parameters

**Figure 2:**
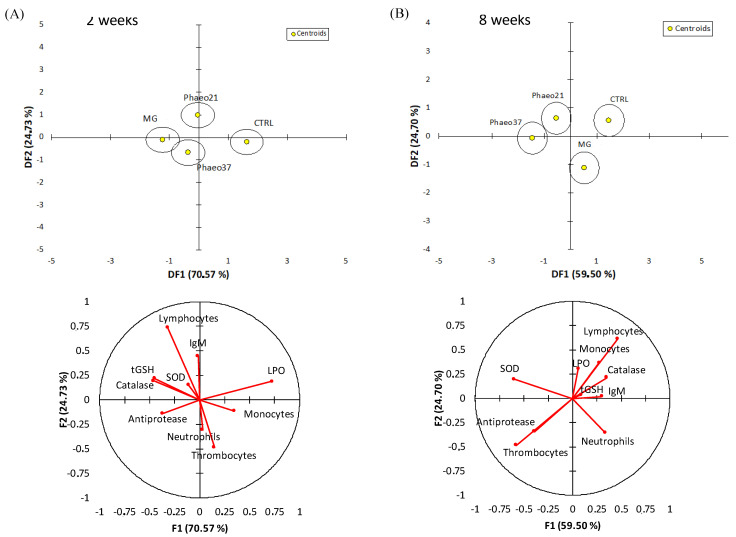
Discriminant analysis of experimental groups based on all physiological biomarkers analysed in the target tissues (yellow marker indicates the centroids of each group) and variables loads for DF1 and DF2 at 2 and 8 weeks. (**A**) 2 weeks; (**B**) 8 weeks.

An overall multivariate analysis combining raw data from haematological, humoral and hepatic oxidative stress biomarkers (using PCA-DA) was performed to discriminate the physiological effects caused by the experimental diets both at 2 and 8 weeks of feeding (Figure 2). The first two discriminant functions accounted for 95% of dataset variability at 2 weeks. Group discrimination was significant (Wilk’s lambda = 0.3, *p* = 0.01) highlighting the differences between CTRL and BG groups (*p* < 0.03). This discrimination was loaded by lower lipid peroxidation, higher CAT and tGSH and to a lesser extent higher antiproteases activity in BG groups. At 8 weeks, groups were discriminated (Wilk’s lambda = 0.2; *p* < 0.001) and the first two discriminant factors accounted for 84% of dataset variability. CTRL and MG dietary treatments were significantly discriminated from the Phaeo groups (*p* < 0.04) and this separation was loaded by a higher SOD activity and thrombocyte percentage in the last groups.

### 2.5. Gene Expression Analysis

**Figure 3:**
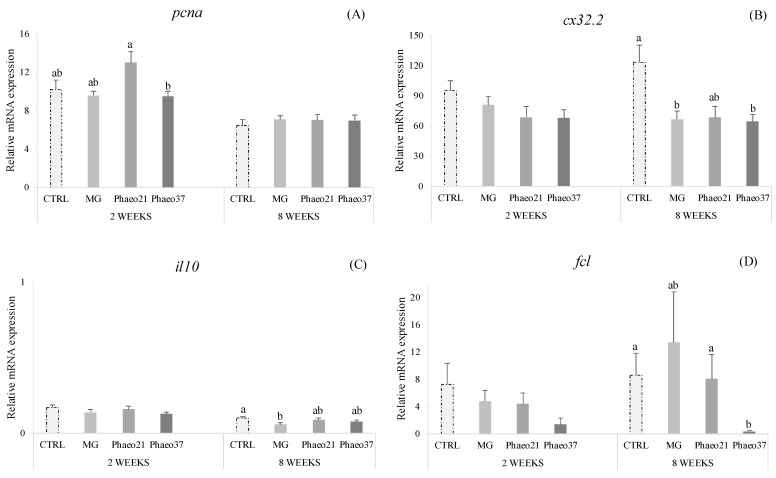
Relative mRNA expression of *pcna* (**A**), *cx32.2* (**B**), *il10* (**C**) and *fcl* (**D**) genes in the anterior intestinal tissue of gilthead seabream juveniles fed the experimental diets for 2 and 8 weeks. Data are the mean ± SEM (*n* = 9). All data values for each gene were in reference to the expression level of *cldn12* of CTRL fish with an arbitrary assigned value of 1. *p* values result from one-way ANOVA. Different lowercase letters indicate significant differences among dietary treatments (*p* < 0.05).

Different pathways represented in this gene array showed significant dietary effects in the proximal intestine. Proliferating cell nuclear antigen gene (*pcna*) was down-regulated in Phaeo37-fed fish at 2 weeks (Figure 3A). In contrast, different genes belonging to different molecular and cellular pathways were down-regulated in seabream-fed Phaeo37 after 8 weeks of feeding, in particular, fucolectin (*fcl*) (Figure 3D), as well as gap junction cx32.2 protein (*cx32.2*) (Figure 3B) genes. Moreover, the latter gene was down-regulated in fish fed MG as well as interleukin 10 (*il10*) (Figure 3C). Complete relative gene expression profile of the anterior intestine is provided as Supplementary Material (Tables S1 and S2). According to the Supplementary Tables, it was found that the mRNA expression levels of the other analysed genes showed no statistically significant differences among experimental groups.

**Figure 4:**
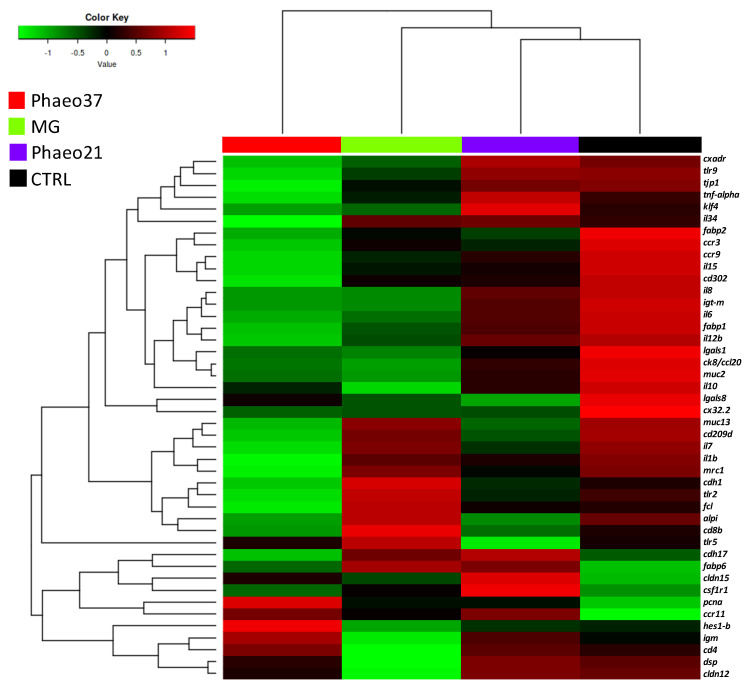
Heat map showing the normalized mRNA levels of selected genes in the anterior intestinal tissue of gilthead seabream juveniles after 8 weeks of feeding. Each block represents the mean mRNA level quantified by qPCR (*n* = 9).

**Figure 5:**
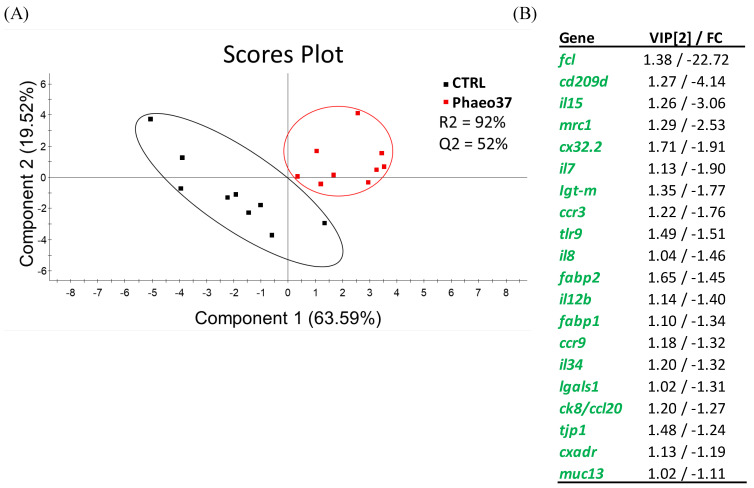
(**A**) Partial least square discriminant analysis (PLS-DA) score plots of all gene expression biomarkers analysed in the proximal intestine of gilthead seabream juveniles along the two main components at 8 weeks. (**B**) Ordered list of markers by variable importance (VIP) in projection of PLS-DA model for group differentiation, as well as the fold-change (FC) in comparison to CTRL. Markers with VIP values > 1 after the first and second main components are represented.

Further differences between fish fed the experimental diets in comparison to CTRL were highlighted by a clustering heatmap of gene expression after 8 weeks of feeding (Figure 4). This approach pointed to Phaeo37 as the experimental diet more apart from CTRL in the gene expression pattern. In order to get a clearer picture of the dietary effect on intestinal gene expression, an overall multivariate analysis combining raw data from the different genes (using PLS-DA) in CTRL and Phaeo 37 groups was performed. For the 2 weeks feeding period, the model was not able to show a clear separation between experimental groups (data not shown). However, at 8 weeks, the same approach showed that expression patterns can be summarized through two main components that explain 88.11% of total variance (Figure 5A). On the one hand, component 1 (63.59% of total variance, X-axis) appeared to be mostly related to diet effect, as it was able to clearly separate Phaeo37- and CTRL-fed fish. On the other hand, component 2 (19.52% of total variance, Y-axis) appeared to account for inner group variability. A total of 20 genes showed a VIP value > 1, highlighting their contribution to diet differences (Figure 5B).

## 3. Discussion

Intensive fish production creates stressful conditions that negatively affect immune function [28], increasing the risk of infection caused by opportunistic bacteria. Therefore, preventive strategies that can improve aquatic animal health and reduce the risk of disease outbreaks must be adopted, such as the use of prebiotics and immunostimulants in feed formulation [29,30,31]. Immunostimulant and prebiotic activities after β-(1,3; 1,6)-glucans administration are well-recognized; thus, these compounds have been suggested as potential nutraceuticals or vaccine adjuvants to enhance immune responses [4,32,33]. For that purpose, in the present study, gilthead seabream juveniles were fed microalgae (*P. tricornutum*) derived BGs in a 2-week cycle pulse-feeding regimen. This nutritional strategy was outlined, as care must be taken not to exhaust the fish immune system due to immunostimulant overexposure after long administration periods [34,35,36,37]. Moreover, intermittent administration seems a suitable approach as BGs apparently can induce long-lived effects in fish [38]. Several studies report increased innate immune parameters and pathogen resistance at least 2 weeks after BGs oral and intra-peritoneal (i.p.) administration [22,39,40].

Studies where BGs were orally administered to fish not only showed immunostimulatory effects but in some cases, improved growth performance [23,24,41,42]. Dawood et al. [43] showed that supplementing red seabream diets up to 0.1% (g/Kg feed) with a commercial BG product (85% purity) for 8 weeks improved final body weight and growth performance as well as lysozyme activity and higher tolerance against a low-salinity stress test when compared with a BG-free fed group. In the current study, β-glucan supplementation did not affect fish growth performance over the course of the trial (8 weeks), independently from its source. However, it did show immunomodulatory effects and improved oxidative stress status in accordance with the findings reported in other studies [23,44,45,46,47]. Dietary treatments appeared to modulate peripheral lymphocyte numbers. Results pointed to an immunostimulatory effect of diet Phaeo21 at 2 weeks feeding, with this particular BG extract apparently affecting the adaptive arm of the immune system with a rise in circulating lymphocytes. Previous works reported increased lymphocyte percentage in comparison to other leucocytes in pompano fish (*Trachinotus Ovatus*) fed 0.05% and 0.10% [45] and Persian sturgeon (*Acipenser persicus*) fed 0.2 and 0.3% yeast BGs [48] for 8 and 6 weeks, respectively. Nonetheless, in the current study, gilthead seabream fed Phaeo21 and MG dietary treatments showed a decrease in circulating lymphocytes percentage compared to those fed CTRL after 8 weeks. Kühlwein, et al. [49] reported no apparent effect on circulating lymphocytes when carp juveniles were fed 0.1, 1 and 2% yeast BGs continuously for 8 weeks.

On the other hand, non-specific humoral parameters (antiprotease, bactericidal activity and circulating IgM) were not affected by the supplementation with 0.06% BGs from *S. cerevisiae* or *P. tricornutum* throughout the experimental period. Accordingly, a study done in gilthead seabream fed a 0.1% supplemented feed with a macroalgae derived BG (laminarin) did not show changes in serum antiprotease activity and IgM levels after 4 weeks of feeding [23]. Yamamoto et al. [50] tested different levels, ranging from 0% to 0.8%, of microalgae (*Euglena gracilis*)-derived BGs in Nile tilapia both in vitro and in vivo. While exposing naïve head-kidney phagocytes directly to BGs facilitated the activation of immune cells increasing bactericidal activity against *Streptococcus iniae* and superoxide anion production, in vivo immune effects were found to be more moderate. Authors reported increased complement system activity but no effects on serum lysozyme and blood leukocytes respiratory burst. Still, previous studies in fish revealed a tendency of BGs oral administration to stimulate or modulate innate immune parameters [21,22,42,44,51,52].

Reactive oxygen species (ROS) are produced as a normal by-product of cellular metabolism but in excess, they can contribute to increased oxidative stress and cause cellular damage. Antioxidant enzymatic machinery is the principal cellular protective mechanism against oxidative stress in fish tissue [53]. BGs are reported to have antioxidant properties and modulate antioxidant enzymes activity as well as inhibiting lipid peroxidation in mammals [54]. In fish, BG injection increased SOD and CAT activities in the intestine [46] and blood erythrocytes [55], suggesting that BGs could improve anti-oxidative capacity. Accordingly, hepatic lipid peroxidation decreased in Phaeo37- and MG-fed animals at an early stage (2 weeks), while hepatic SOD showed a long-term (8 weeks) stimulation pattern, with the Phaeo37-fed group showing the highest activity. Zeng, et al. [46] reported a correlation between higher mRNA transcription of nuclear factor erythroid 2-related factor 2 (Nrf2) gene and increased SOD and CAT genes transcription, which translated in higher enzyme activity in fish injected with a 0.1% BG solution. Nrf proteins, under oxidative conditions translocate to the nucleus where they bind to the antioxidant response element (ARE) [56]. ARE is found in the promoters of several chemoprotective genes, including those involved in the response to oxidative stress [57]. Integrating all physiological responses into a multivariate analysis, dietary effects became clearer and differed between sampling points. At 2 weeks of feeding, all groups received BGs clustered together and were different from CTRL. Differences at this early stage pointed to a dietary effect mainly affecting the antioxidant defences and most prominently decreasing lipid peroxidation corroborating results from the one-way ANOVA. Previous studies reported higher antioxidant enzyme activity and lower lipid peroxidation when fish are previously treated with BGs through different administration routes (i.e., i.p. injection; oral route) [46,55,58]. After a toxicological insult, pre-treated fish with barley-derived BG were able to prevent intestinal Cu-induced lipid peroxidation [46]. Although BGs from the present study differ in origin and solubility, the observed early (2 weeks) beneficial effect was elicited by all BG-supplemented feeds, most likely due to the fact that both soluble and particulate BGs can act as exogenous ROS scavengers. Carballo et al. [59] reported that both a *P. tricornutum* chrysolaminarin-rich extract (soluble) and a yeast BG (particulate) show ROS scavenger activities. At a longer feeding period (8 weeks), the *P. tricornutum*-derived BG-groups showed higher SOD activity and clustered together independently from CTRL and MG. Furthermore, microalgae BG-treated groups also showed higher thrombocyte numbers. Although both Phaeo21 and 37 diets were supplemented with BG enriched extracts, other compounds such as *P. tricornutum* cell wall fragments might be present in the mixture and cannot be ruled out as immunomodulators. *P. tricornutum* cell wall is mainly composed of sulphated polysaccharides [60], which are known to interact with different toll-like receptors (TLRs). These compounds might act as antigens recognized by cell surface receptors activating different leucocyte types. In carp, peripheral thrombocytes constitutively express different TLR genes [61] and have been reported to have phagocytic activity and the ability to ingest particulate antigens possibly acting as an antigen presenting cell [62].

In the present study, at 2 weeks proliferating cell nuclear antigen (*pcna*) gene was down-regulated in the anterior gut of Phaeo37 fed fish. Former studies, report PCNA protein expression inhibition in mammalian cancer cells treated with different glucans including laminarin [63,64]. However, intestinal transcriptional changes were more significant at 8 weeks, where differences between CTRL and Phaeo37 gene profiles can be found. Furthermore, Phaeo37 and MG fed groups showed a down-regulation of different genes when compared to CTRL, namely *il10*, *cx32.2*, *fcl*. Hence, a multivariate analysis was performed (PLS-DA) allowing for a more comprehensive understanding of fish health status, whereas, at the same time identifying the most responsive gene biomarkers in fish intestine. VIP analysis with the first two components, highlighted that the top contributing genes for dietary differences in the gut were immune related (PRR- *fcl, cd209d, mrc1, tlr9, lgals1*; Interleukins- *il7, il8, il12β, il15, il34*; Immunoglobulin production- *igt-m*; chemokines and receptors- *ccr3, ccr9, ck8/cl20*). Phaeo37 dietary treatment caused a general down-regulation of gene transcription. Therefore, the effect of Phaeo37 supplemented diet was mostly immunomodulatory inducing a local anti-inflammatory state at molecular level, which as a consequence led to decreased immune cell activation in the gut. The down-regulation of intestinal immune-related genes can be understood as an immune tolerance effect that can be beneficial in an acute inflammation scenario, counterbalancing its negative and potentially dangerous effects. Falco et al. [25] also found an anti-inflammatory effect in common carp (*Cyprinius carpio L.*) intestine, with several inflammatory genes appearing down regulated when fish were fed a yeast BG supplemented diet for a 2-week period. Furthermore, even after a challenge (i.p. injection) with live bacteria (*Aeromonas salmonicida*), fish fed BGs showed decreased intestinal *il1b*, *il6* and *tnf-alpha* expression, while showing up-regulation in the head-kidney. In this particular case, it seems that BG may be preventing an acute response to infection in the gut, without compromising the systemic response. Additionally, the same down-regulation pattern (inflammatory genes) was seen in the spleen of rainbow trout after 37 days of feeding lentinan (soluble low molecular weight glucan)-supplemented diets [65]. These findings support the idea that BG can have localized specific effects depending on the target tissue.

Overall, some discrepancies can be observed among previous works and data gathered in the present study, which can be explained by different BG preparations. While some studies use crude BG extracts, others use purified compounds differing in molecular weight, branching and solubility. BGs solubility/insolubility seems to play a major role in ligand/receptor recognition and consequently immune cell activation [66,67]. In mammals, particulate BGs directly stimulate immune cell activation through a Dectin-1 recognition pathway, while soluble BG require complement-mediated opsonisation to activate a CR3-dependent pathway [66,68]. Still, in the present work, the particulate BG diet (MG) showed only mild effects mostly related with oxidative defenses after 2 weeks of feeding. In addition to solubility, molecular weight can play an important role in the biological effects of BGs. Different authors have found that in colitis-induced rat models, the dietary administration of low and high molecular weight oat BGs reduced the inflammatory response in colon and also ameliorated the local inflammation [69,70]. However, these authors found that low molecular mass BGs showed a significantly stronger anti-inflammatory effect, through the down-regulation of several pro-inflammatory cytokines and that the therapeutic effect is in evident relation with the molecular mass of the polymer. When comparing the different feeds used in the present study, Phaeo21 and 37 extracts show low molecular mass BGs (chrysolaminarin) [10] while, MG feed is supplemented with a high molecular weight BG (Baker´s yeast) [71,72]. Furthermore, *P. tricornutum* extract supplemented diets although having the same BG concentration, differ in purity, since Phaeo37 extract has a higher percentage of BGs compared to Phaeo21. Thus, the combination of low molecular mass BGs and higher extract purity might explain the higher overall immunomodulatory and oxidative protective effects of Phaeo37 dietary treatment.

In summary, novel feeds with increasingly higher percentages of terrestrial animal- and plant-derived ingredients have been shown to have anti-nutritional factors that often cause gut inflammation in fish, a condition that might lead to impaired nutrient absorption and the disruption of normal microbiota. The use of gut anti-inflammatory compounds can have special relevance nowadays in aquaculture, both as a prophylactic and therapeutic measure, as the industry decreases the use of FM, replacing it by the ingredients referred to above. In this regard, our results indicate that the dietary administration of a *P. tricornutum* 37% enriched-BG extract might be relevant in a context of extreme dietary formulation due to its anti-inflammatory and anti-oxidative effects.

## 4. Materials and Methods

### 4.1. P. tricornutum Extracts

Chrysolaminarin-rich biomass from *P. tricornutum* (SAG 1090-1b) grown under nitrogen-depleted conditions in flat panel airlift reactors was harvested and concentrated via centrifugation to 250–270 g L^−1^ (Clara 20, Alfa Laval, Lund, Sweden). Afterwards, the biomass was frozen at −20 °C. For further processing, the biomass was thawed and diluted to 100 g L^−1^ with deionized water. The cell disruption was performed according to Derwenskus, et al. [73] with a ball mill (PML-2, Bühler, Uzwil, Switzerland). Phaeo21 was freeze dried after cell disruption (VaCo 5, Zirbus, Bad Grund, Germany), while Phaeo37 was centrifuged and the supernatant freeze-dried (Avanti J-26 XP, Beckman Coulter, Brea, CA, USA).

### 4.2. Diet Composition

The trial comprised four isonitrogenous (63% crude protein) and isolipidic (17% crude fat) diets (Table 4). A high-quality, practical diet was used as control (CTRL) and 3 experimental diets based on CTRL were supplemented with either a commercial product derived from *S. cerevisiae* (diet MG) or different extracts of *P. tricornutum* (diets Phaeo21 and Phaeo37), to obtain a final concentration of 0.6 g β-glucans per Kg of feed (0.06%) in all supplemented diets. Diets were manufactured by SPAROS. All powder ingredients were mixed according to the target formulation in a double-helix mixer (model RM90, MAINCA, Barcelona, Spain) and ground (below 200 µm) in a micropulverizer hammer mill (model SH1, Hosokawa-Alpine, Augsburg, Germany). Subsequently, the oils were added to the mixtures, which were humidified with 20–25% water and agglomerated by a low-shear and low-temperature extrusion process (ITALPLAST, West Heidelberg, VIC, Australia). Extruded pellets (1.5 mm) were dried in a vibrating fluid bed dryer (model DR100, TGC Extrusion, Roullet-Saint-Estèphe, France). Diets were packed in sealed plastic buckets and shipped to the research site (Riasearch, Murtosa, Portugal) where they were stored at room temperature in a cool and aerated emplacement. Samples of each diet were taken for analytical characterization.

### 4.3. Fish Rearing Conditions and Feeding Trial

**Figure 6:**
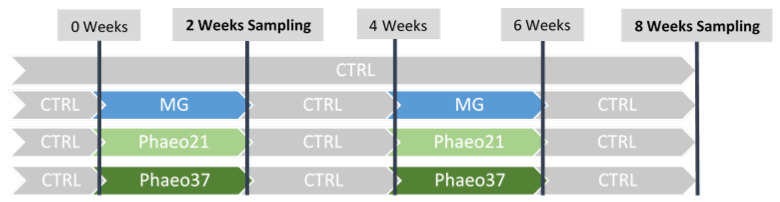
Schematic overview of the experimental design.

Fish were reared in a seawater recirculation system with aeration (mean dissolved oxygen above 6 mg L^−1^) and water flow at 3 L min^−1^ (mean temperature 24.1 ± 0.6 °C; mean salinity 18.7 ± 0.1‰). Water quality parameters were monitored daily (mean dissolved oxygen 6.4 ± 1.0 mg L^−1^; mean unionized ammonia levels 0.001 ± 0.002 mg L^−1^). Diets were randomly assigned to quadruplicate groups of 95 gilthead seabream juveniles (initial body weight: 4.1 ± 0.1 g) that were fed to satiation three times a day for 8 weeks in a pulse-feeding regimen. Accordingly, in fish fed the different experimental diets, the CTRL diet was intercalated every 2 weeks, as shown in Figure 6.

### 4.4. Sampling Procedures

Fish were individually weighed at the beginning and after 2 and 8 weeks of the feeding trial and feed consumption for each experimental replicate was registered daily. After 2 and 8 weeks, three fish per tank were euthanized with a 2-phenoxyethanol lethal dose (0.5 mL L^−1^) [74], weighed and sampled for tissues (blood, head-kidney, liver and gut). Blood was collected from the caudal vein using heparinized syringes and centrifuged at 10,000× *g* during 10 min at 4 °C to obtain plasma samples. Plasma, head-kidney and liver samples were immediately frozen at −80 °C, and anterior intestine was preserved in RNA later until further analysis.

### 4.5. Haematological Procedures

Blood smears were prepared from peripheral blood, air dried and stained with Wright’s stain (Haemacolor; Merck) after fixation for 1 min with formol–ethanol (10% formaldehyde in ethanol). Neutrophils were labelled through the detection of peroxidase activity revealed by the Antonow´s technique described by Afonso, et al. [75]. The slides were examined under oil immersion (1000×), and at least 200 leucocytes were counted and classified as thrombocytes, lymphocytes, monocytes and neutrophils. The relative percentage of each cell type was calculated.

### 4.6. Innate Humoral Parameters

Plasma bactericidal activity was determined following the method described by Machado, et al. [76] with some modifications. *Edwardsiella tarda* (*E. tarda*) strain ACC 53.1, gently provided by Prof. Alicia Toranzo (University of Santiago, Spain) was used in the protocol. Briefly, 20 μL of plasma were mixed with 20 μL of bacteria suspension (10^8^ CFU mL^−1^) in duplicate in a flat-bottom 96-well plate that was incubated for 2.5 h at 25 °C (positive control: 20 μL of TSB instead of plasma). Afterwards, 25 μL of 3-(4,5 dimethyl-2-yl)-2,5-diphenyl tetrazolium bromide (1 mg mL^−1^; Sigma, St. Louis, MO, USA) was added to each well and incubated for 10 min at 25 °C to allow the formation of formazan precipitates. Plates were then centrifuged at 2000× *g* for 10 min, the supernatant was discarded and the precipitate was dissolved in 200 μL of dimethyl sulfoxide (Sigma, St. Louis, MO, USA). The absorbance was then measured at 560 nm. Bactericidal activity is expressed as a percentage calculated from the difference between surviving bacteria compared to the number of bacteria from positive controls (100%).

Anti-protease activity was determined as described by Ellis et al. [77] with some modifications. Briefly, 10 µL of plasma were incubated with the same volume of trypsin solution (5 mg mL^−1^ in NaHCO_3_, 5 mg mL^−1^, pH 8.3) for 10 min at 22 °C. After incubation, 100 µL of phosphate buffer (NaH_2_PO_4_, 13.9 mg mL^−1^, pH 7.0) and 125 μL of azocasein solution (20 mg mL^−1^ in NaHCO_3_, 5 mg mL^−1^, pH 8.3) were added and incubated for 1 h at 22 °C. Finally, 250 μL of trichloroacetic acid were added to the reaction mixture and incubated for 30 min at 22 °C. The mixture was centrifuged at 10,000× *g* for 5 min at room temperature. Afterwards, 100 μL of the supernatant was transferred to a 96-well plate and mixed with 100 μL of NaOH (40 mg mL^−1^). The OD was read at 450 nm in a Synergy HT microplate reader. Phosphate buffer instead of plasma and trypsin served as blank, whereas the reference sample was phosphate buffer instead of plasma. The sample inhibition percentage of trypsin activity was calculated as follows: 100 − ((sample absorbance/reference absorbance) × 100). All analyses were conducted in duplicate.

Plasma immunoglobulins (IgMs) were measured by an ELISA assay. Briefly, plasma samples were diluted (1:100) in Na_2_CO_3_ (50 mM, pH = 9.6). Diluted plasma samples (100 μL in duplicate) were incubated overnight (4 °C) in a 96 well plate, using Na_2_CO_3_ (100 μL) as a negative control. The samples (antigen) were then removed and 300 μL of blocking buffer (5% low fat milk in 0.1% Tween 20) was added to each well and incubated for 1 h at 22 °C. This mixture was then removed, followed by three consecutive washes with 300 μL of T-TBS (0.1% Tween 20). After properly cleaning and drying the wells, 100 μL of the anti-seabream primary IgM monoclonal antibody (1:200 dilution in blocking buffer; Aquatic Diagnostics, UK) was added to each well and incubated for 1 h at 22 °C. The primary antibody was then removed by aspiration, with three consecutive washes being performed. Afterwards, the anti-mouse IgG-HRP, secondary antibody (1:1000 dilution in blocking buffer; SIGMA), was added and incubated for 1 h at 22 °C, then removed by aspiration. The wells were again washed three times and 100 μL of TMB substrate solution for ELISA (BioLegend #421101), was added to each well and incubated for 5 min. The reaction was stopped after 5 min by adding 100 μL of H_2_SO_4_ 2 M and the optical density was read at 450 nm.

### 4.7. Analysis of Oxidative Stress Biomarkers

Liver samples were thawed and homogenized (1:10) in phosphate buffer 0.1 M (pH 7.4) using Precellys evolution tissue lyser homogenizer.

One aliquot of tissue homogenate was used to determine the extent of endogenous lipid peroxidation (LPO) by measuring thiobarbituric acid-reactive species (TBARS) as suggested by Bird and Draper [78]. To prevent artifactual lipid peroxidation, butylhydroxytoluene (BHT 0.2 mM) was added to the aliquot. Briefly, 1 mL of 100% trichloroacetic acid and 1 mL of 0.73% thiobarbituric acid solution (in Tris–HCl 60 mM pH 7.4 with DTPA 0.1 mM) were added to 0.2 mL of liver homogenate. After incubation at 100 °C for 60 min, the solution was centrifuged at 12,000× *g* for 5 min and LPO levels were determined at 535 nm.

The remaining tissue homogenate was centrifuged for 20 min at 10,000× *g* (4 °C) to obtain the post mitochondrial supernatant fraction (PMS). Total proteins in homogenates were measured by using Pierce™ BCA Protein Assay Kit, as described by the manufacturer.

Catalase (CAT) activity was determined in PMS by measuring substrate (H_2_O_2_) consumption at 240 nm according to Claiborne [79] adapted to microplate. Briefly, in a microplate well, 0.140 mL of phosphate buffer (0.05 M pH 7.0) and 0.150 mL H_2_O_2_ solution (30 mM in phosphate buffer 0.05 M pH 7.0) were added to 0.01 mL of liver PMS (0.7 mg ml^−1^ total protein). Enzymatic activity was determined in a microplate reader (BioTek Synergy HT) reading the optical density at 240 nm for 2 min every 15 sec interval.

Superoxide dismutase (SOD) activity was measured according to Flohé and Otting [80] adapted to microplate by Lima, et al. [81]. Briefly, in a microplate well, 0.2 mL of the reaction solution [1 part xantine solution 0.7 mM (in NaOH 1 mM) and 10 parts cytochrome c solution 0.03 mM (in phosphate buffer 50 mM pH 7.8 with 1 mM Na-EDTA)] was added to 0.05 mL of liver PMS (0.25 mg ml^−1^ total protein). Optical density was measured at 550 nm in a microplate reader (BioTek Synergy HT, Winooski, VT, USA) every 20-s interval for 3 min at 25° C.

Total glutathione (tGSH) content was determined with PMS fraction at 412 nm using a recycling reaction of reduced glutathione (GSH) with 5,5-dithiobis-(2-nitrobenzoic acid) (DTNB) in the presence of glutathione reductase (GR) excess [82,83]. TG content is calculated as the rate of TNB^2-^ formation with an extinction coefficient of DTNB chromophore formed, ε = 14.1 × 10^3^ M^−1^cm^−1^.

### 4.8. Gene Expression

Total RNA isolation from target tissue (anterior intestine) was conducted with NZY Total RNA Isolation kit (NZYTech, Lisbon, Portugal) following the manufacturer’s specifications. Reverse transcription (RT) of 500 ng total RNA was performed with random decamers using a High-Capacity cDNA Reverse Transcription Kit (Applied Biosystems, Foster City, CA, USA) according to the manufacturer’s instructions. RT reactions were incubated for 10 min at 25 °C and 2 h at 37 °C. Negative control reactions were run without reverse transcriptase. Real-time quantitative PCR was carried out on a EpMotion 5070 Liquid Handling Robot (Eppendorf, Hamburg, Germany) using a 96-well PCR array layout with 44 genes designed for simultaneously profiling of anterior intestine (Table 5). Genes comprised in the array were selected for their involvement in gut integrity, health, immunity and signal transduction. Specific primer pair sequences are listed in Table S3. Controls of general PCR performance were included on each array, and all the pipetting operations performed by means of the EpMotion 5070 Liquid Handling Robot (Eppendorf, Hamburg, Germany). Briefly, RT reactions were diluted to obtain the equivalent concentration of 660 pg of total input RNA which were used in a 25-µL volume for each PCR reaction. PCR wells contained a 2× SYBR Green Master Mix (Bio-Rad) and specific primers at a final concentration of 0.9 μM were used to obtain amplicons 50–150 bp in length. The program used for PCR amplification included an initial denaturation step at 95 °C for 3 min, followed by 40 cycles of denaturation for 15 s at 95 °C and annealing/extension for 60 s at 60 °C. The efficiency of PCR reactions was always higher than 90%, and negative controls without sample templates were routinely performed for each primer set. The specificity of reactions was verified by analysis of melting curves (ramping rates of 0.5 °C/10 s over a temperature range of 55–95 °C), and linearity of serial dilutions of RT reactions. Fluorescence data acquired during the PCR extension phase were normalized using the delta–delta Ct method [84]. Beta-actin (*actb*) was tested for gene expression stability using GeNorm software (M score = 0.13) and it was used as housekeeping gene in the normalization procedure. For comparing the mRNA expression level of a panel of genes in a given dietary treatment, all data values were in reference to the expression level of claudin 12 (*cldn12*) in CTRL fish, which was arbitrarily assigned a value of 1.

### 4.9. Data Analysis

All results are expressed as mean ± standard error (mean ± SE). Residuals were tested for normality (Shapiro–Wilk´s test) and homogeneity of variance (Levene’s test). When residuals did not meet the assumptions, data were transformed by a Box-Cox transformation. One-way ANOVAs were performed for all datasets, with “dietary treatment” as the fixed effect, followed by multiple comparison Tukey post-hoc tests. The factor “time” was not considered in the analysis since it is not a goal of the study to evaluate how time affects the measured variables, and groups were treated independently for 2 and 8 weeks.

In an attempt to discriminate and classify individuals by the existing groups, a multivariate canonical discriminant analysis was performed on the physiological dataset (obtained from blood, plasma and liver tissues analyses) to evaluate the linear combinations of the original variables that will best separate the groups (discriminant functions). Each discriminant function explains part of total variance of the dataset and is loaded by variables contributing the most for that variation. Discriminatory effectiveness was assessed by Wilk’s λ test, and the distance between groups’ centroids was measured by squared Mahalanobis distance, and Fisher’s F statistic was applied to infer significance. All statistical analyses were performed using the computer package SPSS 26 for WINDOWS.

Gene expression results were evaluated with an unsupervised multivariate analysis by principal component analysis (PCA) as an unbiased statistical method to observe intrinsic trends in the dataset, using EZ-INFO^®^ v3.0 (Umetrics, Sweden). To achieve the maximum separation among the groups, a supervised multivariate analysis by partial least-squares discriminant analysis (PLS-DA) was sequentially applied, using EZ-INFO^®^ v3.0 (Umetrics, Umeå, Sweden). Potential differential genes were selected according to the Variable Importance in the Projection (VIP) values. Variables with VIP > 1 were considered to be influential for the separation of samples in PLS-DA analysis [85,86,87]. The level of significance used was *p* ≤ 0.05 for all statistical tests. Heat map of transcript levels were produced with the R package gplots, using the average linkage method and Euclidean distance.

## Figures and Tables

**Table 1:** Growth performance parameters in gilthead seabream juveniles after 8 weeks of feeding regimen. Data are the mean ± SEM (*n* = 4).

	8 Weeks			
DIETS	CTRL	MG	Phaeo21	Phaeo37
IBW	4.18 ± 0.04	4.12 ± 0.02	4.12 ± 0.05	4.15 ± 0.05
FBW	41.36 ± 0.81	42.48 ± 0.43	42.08 ± 0.53	41.93 ± 0.97
RGR	3.77 ± 0.03	3.83 ± 0.02	3.82 ± 0.03	3.8 ± 0.03
FCR	1.20 ± 0.01	1.19 ± 0.01	1.21 ± 0.02	1.19 ± 0.04

IBW: initial body weight (g); FBW: final body weight (g); RGR: relative growth rate (% average body weight/day) and FCR: feed conversion ratio.

**Table 2:** Percentage values of peripheral blood leucocytes (thrombocytes, lymphocytes, monocytes and neutrophils) in gilthead seabream juveniles after 2 and 8 weeks of feeding regimen. Data are the mean ± SEM (*n* = 12).

	2 Weeks				8 Weeks			
DIETS	CTRL	MG	Phaeo21	Phaeo37	CTRL	MG	Phaeo21	Phaeo37
CELLS (%)								
Thrombocytes	65.2 ± 2.0	63.0 ± 2.2	60.3 ± 1.8	64.7 ± 1.6	71.2 ^b^ ± 2.6	78.5 ^a^ ± 1.4	76.4 ^a,b^ ± 2.5	81.0 ^a^ ± 0.8
Lymphocytes	24.1 ^b^ ± 1.5	28.3 ^a,b^ ± 1.9	30.7 ^a^ ± 1.4	24.8 ^b^ ± 1.3	18.4 ^a^ ± 2.5	13.0 ^b^ ± 1.0	13.1 ^b^ ± 2.6	13.4 ^a,b^ ± 0.8
Monocytes	5.3 ± 0.8	3.5 ± 0.7	4.4 ± 0.7	5.2 ± 0.4	3.4 ± 0.6	2.4 ± 0.7	3.1 ± 0.6	2.0 ± 0.5
Neutrophils	4.4 ± 1.0	4.1 ± 0.9	4.1 ± 0.7	4.8 ± 0.7	4.7 ± 0.4	5.6 ± 0.6	4.3 ± 0.7	3.6 ± 0.6

Different superscript letters indicate significant differences between diets (*p* < 0.05) within the same sampling point.

**Table 3:** Plasma immune parameters of gilthead seabream juveniles after 2 and 8 weeks feeding (antiprotease activity, bactericidal activity and immunoglobulin M). Data are the mean ± SEM (*n* = 12). Different letters indicate significant differences between dietary treatments (*p* < 0.05).

	2 Weeks				8 Weeks			
DIETS	CTRL	MG	Phaeo21	Phaeo37	CTRL	MG	Phaeo21	Phaeo37
Antiprotease act (%)	95.7 ± 0.6	96.0 ± 0.5	95.5 ± 0.7	95.4 ± 0.6	97.9 ± 0.2	98.1 ± 0.1	97.9 ± 0.2	98.2 ± 0.2
Bactericidal act (%)	45.0 ± 6.3	35.5 ± 4.0	40.3 ± 6.6	45.4 ± 4.4	53.3 ± 7.2	56.5 ± 5.8	57.4 ± 6.8	61.8 ± 4.8
IgM (OD 450 nm)	0.31 ± 0.03	0.32 ± 0.03	0.37 ± 0.05	0.29 ± 0.03	0.62 ± 0.05	0.53 ± 0.03	0.47 ± 0.05	0.55 ± 0.03

**Table 4:** Ingredients and proximate composition of experimental diets.

Ingredients %	CTRL	MG	Phaeo21	Phaeo37
Fishmeal ^1^	20.00	20.00	20.00	20.00
Fish protein hydrolysate ^2^	8.00	8.00	8.00	8.00
Squid meal ^3^	21.00	21.00	21.00	21.00
Krill meal ^4^	16.50	16.50	16.50	16.50
Wheat gluten ^5^	11.50	11.50	11.50	11.50
Wheat meal ^6^	0.29	0.19		0.13
Vitamin and mineral premix ^7^	2.00	2.00	2.00	2.00
Lecithin ^8^	4.30	4.30	4.30	4.30
Fish oil ^9^	6.50	6.50	6.50	6.50
Binders, antioxidant and other additives ^10^	9.91	9.91	9.91	9.91
Yeast beta-glucans ^11^		0.10		
Algae beta-glucans Phaeo21 ^12^			0.29	
Algae beta-glucans Phaeo37 ^13^				0.16
Proximate composition				
Dry matter (DM) %	94.60	94.20	94.20	94.50
Ash, % DM	9.60	9.50	9.50	9.50
Crude protein, % DM	62.90	62.80	62.80	62.90
Crude fat, % DM	17.10	17.10	17.10	17.10
Gross energy (kJ g^−1^ DM)	22.90	22.90	22.90	22.90

^1^ Super Prime: 66.3% CP, 11.5% CF, Pesquera Diamante, Peru; ^2^ CPSP 90: 82% CP 9% CF, Sopropêche, France; ^3^ Squid meal without guts: 83% CP, 4% CF, Sopropêche, France; ^4^ Krill meal: 61.1% CP, 17.4% CF, Aker Biomarine, Norway; ^5^ VITEN: 82% CP, 2.1% CF, Roquette, France; ^6^ Wheat meal: 10.2% CP; 1.2% CF, MOLISUR, Spain; ^7^ PREMIX Lda, Portugal: Vitamins (IU or mg/kg diet): DL-alpha tocopherol acetate, 200 mg; sodium menadione bisulphate, 50 mg; retinyl acetate, 40,000 IU; DL-cholecalciferol, 4000 IU; thiamin, 60 mg; riboflavin, 60 mg; pyridoxine, 40 mg; cyanocobalamin, 0.2 mg; nicotinic acid, 400 mg; folic acid, 30 mg; ascorbic acid, 1000 mg; inositol, 1000 mg; biotin, 6 mg; calcium panthotenate, 200 mg; choline chloride, 2000 mg, betaine, 1000 mg. Minerals (g or mg/kg diet): copper sulphate, 18 mg; ferric sulphate, 12 mg; potassium iodide, 1 mg; manganese oxide, 20 mg; sodium selenite, 0.02 mg; zinc sulphate, 27.5 mg; sodium chloride, 800 mg; excipient wheat middling’s; ^8^ LECICO GmbH, Germany; ^9^ Sopropêche, France; ^10^ Confidential blend of constant binders and other additives; ^11^ Macrogard, 67.2% beta-glucans, Biorigin, Brazil; ^12^ Beta-glucan rich biomass of microalgae (*Phaeodactylum tricornutum* from SAG culture collection) with 21% beta-glucans; ^13^ Beta-glucan rich extract of microalgae (*Phaeodactylum tricornutum* from SAG culture collection) with 37% beta-glucans.

**Table 5:** PCR-array layout for gene expression profiling of anterior intestine in seabream.

Function	Gene	Symbol	GenBank
Epithelia integrity	proliferating cell nuclear antigen	*pcna*	KF857335
	transcription factor HES-1-B	*hes1-b*	KF857344
	krueppel-like factor 4	*klf4*	KF857346
	claudin-12	*cldn12*	KF861992
	claudin-15	*cldn15*	KF861993
	cadherin-1	*cdh1*	KF861995
	cadherin-17	*cdh17*	KF861996
	tight junction protein ZO-1	*tjp1*	KF861994
	desmoplakin	*dsp*	KF861999
	gap junction Cx32.2 protein	*cx32.2*	KF862000
	coxsackievirus and adenovirus receptor homolog	*cxadr*	KF861998
Nutrient transport	intestinal-type alkaline phosphatase	*alpi*	KF857309
	liver type fatty acid-binding protein	*fabp1*	KF857311
	intestinal fatty acid-binding protein	*fabp2*	KF857310
	ileal fatty acid-binding protein	*fabp6*	KF857312
Mucus production	mucin 2	*muc2*	JQ277710
	mucin 13	*muc13*	JQ277713
Interleukins	tumor necrosis factor-alpha	*tnf-alpha*	AJ413189
	interleukin 1 beta	*il1b*	AJ419178
	interleukin 6	*il6*	EU244588
	interleukin 7	*il7*	JX976618
	interleukin 8	*il8*	JX976619
	interleukin 10	*il10*	JX976621
	interleukin 12 subunit beta	*il12b*	JX976624
	interleukin 15	*il15*	JX976625
	interleukin 34	*il34*	JX976629
Cell markers	cluster differentiation 4	*cd4*	AM489485
	cluster differentiation 8 beta	*cd8b*	KX231275
	C-C chemokine receptor 3	*ccr3*	KF857317
	C-C chemokine receptor 9	*ccr9*	KF857318
	C-C chemokine receptor 11	*ccr11*	KF857319
	C-C chemokine ck8/C-C motif chemokine ligand 20	*ck8/ ccl20*	GU181393
	macrophage colony-stimulating factor 1 receptor	*csf1r*	AM050293
Ig production	immunoglobulin M	*igm*	JQ811851
	immunoglobulin T membrane-bound form	*igt-m*	KX599201
Pathogen associated	galectin 1	*lgals1*	KF862003
microbial pattern	galectin 8	*lgals8*	KF862004
(PAMP)	toll like receptor 2	*tlr2*	KF857323
	toll like receptor 5	*tlr5*	KF857324
	toll like receptor 9	*tlr9*	AY751797
	CD209 antigen-like protein D	*cd209d*	KF857327
	CD302 antigen	*cd302*	KF857328
	macrophage mannose receptor 1	*mrc1*	KF857326
	fucolectin	*fcl*	KF857331

## Data Availability

Not applicable.

## Supplementary Materials

The following are available online at https://www.mdpi.com/article/10.3390/md19120653/s1, Table S1. Relative gene expression profiling of anterior intestine in gilthead seabream juveniles fed experimental diets for 2 weeks; Table S2. Relative gene expression profiling of anterior intestine in gilthead seabream juveniles fed experimental diets for 8 weeks; Table S3: Primers for qPCR amplification in seabream.
