# Supplementary material for: Immune Status and Hepatic Antioxidant Capacity of Gilthead Seabream Sparus aurata Juveniles Fed Yeast and Microalga Derived β-glucans
Source: Mar Drugs. 2021 Nov 23;19(12):653. doi: 10.3390/md19120653 (PMC8704051; doi:10.3390/md19120653)
Supplement: Supplementary file 1 [file marinedrugs-19-00653-s001.zip › Reis et al_2021_suplemmentary material_Final version_15112021.pdf]

Table S1. Relative gene expression profiling of anterior intestine in gilthead seabream juveniles fed experimental diets for 2 weeks. Data are the mean ± SEM (n=9). All data values for each tissue were in reference to the expression level of *cldn12* of CTRL fish with an arbitrary assigned value of 1.

	CTRL	MG	Phaeo21	Phaeo37	*P*-value[1]
pcna	10.22 ± 0.97[ab]	9.59 ± 0.45[ab]	12.99 ± 1.14[a]	9.49 ± 0.56[b]	**0.028**
hes1-b	2.37 ± 0.30	1.86 ± 0.17	2.17 ± 0.18	1.88 ± 0.13	0.287
klf4	3.48 ± 0.23	4.24 ± 0.59	2.91 ± 0.29	3.03 ± 0.14	0.201
cldn12	1.00 ± 0.04	0.98 ± 0.10	1.01 ± 0.06	0.96 ± 0.05	0.350
cldn15	17.96 ± 1.81	15.40 ± 1.75	18.87 ± 1.28	15.33 ± 1.22	0.215
cdh1	20.23 ± 0.96	19.45 ± 1.14	19.24 ± 1.13	18.33 ± 1.03	0.647
cdh17	90.58 ± 4.54	84.13 ± 4.45	89.38 ± 3.25	77.46 ± 2.93	0.084
tjp1	0.76 ± 0.03	0.74 ± 0.04	0.74 ± 0.03	0.76 ± 0.04	0.942
dsp	7.51 ± 0.42	7.12 ± 0.64	8.10 ± 0.41	6.35 ± 0.28	0.060
cx32.2	95.30 ± 9.78	80.92 ± 8.33	68.93 ± 10.83	68.20 ± 8.00	0.160
cxadr	5.02 ± 0.34	4.25 ± 0.46	4.66 ± 0.24	4.00 ± 0.16	0.108
alpi	67.89 ± 9.78	61.19 ± 10.40	55.41 ± 9.12	65.75 ± 10.49	0.777
fabp1	119.10 ± 7.40	112.74 ± 11.75	109.79 ± 5.30	101.19 ± 6.01	0.211
fabp2	835.42 ± 131.39	772.79 ± 119.22	891.79 ± 119.74	702.29 ± 71.58	0.780
fabp6	0.13 ± 0.06	0.03 ± 0.01	0.03 ± 0.01	0.02 ± 0.01	0.863
muc2	36.90 ± 2.25	34.90 ± 1.36	37.06 ± 3.94	31.92 ± 4.55	0.456
muc13	117.68 ± 7.82	101.79 ± 8.13	114.59 ± 9.31	104.98 ± 3.14	0.423
tnf-alpha	0.21 ± 0.02	0.21 ± 0.02	0.18 ± 0.02	0.18 ± 0.02	0.396
il1b	0.18 ± 0.02	0.19 ± 0.02	0.15 ± 0.01	0.18 ± 0.02	0.471
il6	0.03 ± 0.00	0.04 ± 0.01	0.03 ± 0.00	0.03 ± 0.00	0.227
il7	0.82 ± 0.07	0.93 ± 0.07	0.84 ± 0.06	0.88 ± 0.05	0.634
il8	0.22 ± 0.02	0.27 ± 0.03	0.26 ± 0.03	0.24 ± 0.03	0.440
il10	0.17 ± 0.02	0.14 ± 0.02	0.16 ± 0.02	0.13 ± 0.01	0.264
il12b	0.63 ± 0.05	0.59 ± 0.05	0.68 ± 0.08	0.53 ± 0.04	0.277
il15	0.59 ± 0.03	0.60 ± 0.05	0.69 ± 0.04	0.60 ± 0.03	0.295
il34	2.66 ± 0.12	2.62 ± 0.12	2.45 ± 0.11	2.71 ± 0.13	0.491
cd4-full	0.59 ± 0.11	0.54 ± 0.08	0.56 ± 0.05	0.52 ± 0.07	0.925
cd8b	0.06 ± 0.01	0.09 ± 0.01	0.07 ± 0.01	0.06 ± 0.00	0.431
ccr3	1.68 ± 0.17	1.84 ± 0.25	1.39 ± 0.08	1.72 ± 0.14	0.292
ccr9	1.21 ± 0.10	1.24 ± 0.12	1.14 ± 0.06	1.17 ± 0.10	0.962
ccr11	4.65 ± 0.72	3.31 ± 0.41	4.34 ± 0.50	3.25 ± 0.25	0.125
ck8 / ccl20	6.57 ± 0.43	6.94 ± 0.67	8.15 ± 0.96	7.65 ± 0.37	0.414
csf1r1	1.06 ± 0.07	1.15 ± 0.08	1.03 ± 0.09	0.95 ± 0.06	0.308
Igm	3.77 ± 0.66	4.05 ± 0.74	3.67 ± 0.94	3.92 ± 1.24	0.928
igt-m	0.36 ± 0.04	0.37 ± 0.06	0.39 ± 0.07	0.29 ± 0.05	0.409
lgals1	13.55 ± 1.58	12.45 ± 1.51	13.27 ± 1.59	11.60 ± 1.20	0.762
lgals8	2.84 ± 0.16	2.50 ± 0.18	2.94 ± 0.26	2.66 ± 0.18	0.420
tlr2	0.54 ± 0.04	0.47 ± 0.03	0.48 ± 0.03	0.45 ± 0.03	0.349
tlr5	0.06 ± 0.01	0.05 ± 0.01	0.06 ± 0.01	0.04 ± 0.00	0.148
tlr9	0.06 ± 0.01	0.06 ± 0.01	0.06 ± 0.01	0.05 ± 0.01	0.881
cd209d	0.16 ± 0.02	0.15 ± 0.03	0.12 ± 0.02	0.13 ± 0.02	0.724
cd302	8.40 ± 0.32	7.91 ± 0.43	8.41 ± 0.58	7.53 ± 0.43	0.437
mrc1	1.60 ± 0.09	1.94 ± 0.27	1.64 ± 0.11	1.63 ± 0.10	0.738
fcl	7.32 ± 3.06	4.82 ± 1.59	4.41 ± 1.59	1.38 ± 0.97	0.107

[1]*P* values result from one-way ANOVA. Different superscript letters in each row indicate significant differences among dietary treatments (Tukey post-hoc test $P < 0.05$).

Table S2. Relative gene expression profiling of anterior intestine in gilthead seabream juveniles fed experimental diets for 8 weeks. Data are the mean ± SEM (n=9). All data values for each tissue were in reference to the expression level of *cldn12* of CTRL fish with an arbitrary assigned value of 1.

	**CTRL**	**MG**	**Phaeo21**	**Phaeo37**	***P*-value[1]**
pcna	6.48 ± 0.58	7.08 ± 0.42	7.01 ± 0.59	6.99 ± 0.59	0.788
hes1-b	0.71 ± 0.17	0.63 ± 0.04	0.70 ± 0.05	1.02 ± 0.23	0.402
klf4	2.40 ± 0.23	2.17 ± 0.21	2.70 ± 0.35	2.08 ± 0.15	0.448
cldn12	0.92 ± 0.08	0.85 ± 0.07	0.93 ± 0.08	0.91 ± 0.06	0.868
cldn15	36.90 ± 3.93	39.04 ± 3.47	44.59 ± 3.52	42.56 ± 2.86	0.397
cdh1	21.37 ± 1.03	22.56 ± 0.83	20.94 ± 1.56	19.93 ± 1.14	0.471
cdh17	67.56 ± 2.31	69.25 ± 2.03	69.82 ± 4.91	66.67 ± 3.73	0.937
tjp1	0.50 ± 0.02	0.47 ± 0.03	0.50 ± 0.05	0.41 ± 0.03	0.202
dsp	4.31 ± 0.28	3.58 ± 0.27	4.38 ± 0.44	4.20 ± 0.34	0.308
cx32.2	123.59 ± 16.89[a]	66.78 ± 8.39[b]	68.64 ± 10.75[ab]	64.56 ± 6.85[b]	**0.034**
cxadr	5.02 ± 0.41	4.46 ± 0.46	5.16 ± 0.43	4.23 ± 0.39	0.296
alpi	79.98 ± 11.58	83.98 ± 12.39	68.88 ± 10.80	67.95 ± 7.74	0.798
fabp1	220.17 ± 24.84	180.66 ± 15.00	203.05 ± 16.45	164.57 ± 14.70	0.162
fabp2	1144.00 ± 114.86	931.28 ± 93.59	879.36 ± 61.09	786.87 ± 67.85	0.094
fabp6	0.03 ± 0.01	0.05 ± 0.02	0.04 ± 0.02	0.03 ± 0.01	**0.049**
muc2	21.84 ± 2.71	17.65 ± 2.57	19.77 ± 4.03	18.13 ± 2.64	0.737
muc13	127.18 ± 5.91	126.41 ± 7.70	117.73 ± 8.32	114.57 ± 9.25	0.516
tnf-alpha	0.19 ± 0.03	0.19 ± 0.01	0.21 ± 0.02	0.17 ± 0.02	0.534
il1b	0.13 ± 0.02	0.13 ± 0.01	0.12 ± 0.02	0.10 ± 0.01	0.628
il6	0.02 ± 0.00	0.02 ± 0.00	0.02 ± 0.00	0.02 ± 0.00	0.461
il7	2.58 ± 0.72	2.50 ± 0.99	1.94 ± 0.56	1.35 ± 0.41	0.558
il8	0.24 ± 0.05	0.17 ± 0.03	0.22 ± 0.04	0.17 ± 0.02	0.584
il10	0.10 [a] ± 0.01	0.06 [b] ± 0.01	0.09 [ab] ± 0.01	0.08 [ab] ± 0.01	**0.042**
il12b	0.59 ± 0.08	0.48 ± 0.04	0.56 ± 0.04	0.42 ± 0.04	0.107
il15	4.06 ± 1.26	2.57 ± 1.19	2.84 ± 0.81	1.33 ± 0.37	0.324
il34	3.65 ± 0.39	3.78 ± 0.59	3.82 ± 0.33	2.77 ± 0.30	0.186
cd4-full	0.34 ± 0.02	0.28 ± 0.02	0.35 ± 0.04	0.35 ± 0.04	0.406
cd8b	0.09 ± 0.01	0.10 ± 0.03	0.08 ± 0.01	0.08 ± 0.01	0.906
ccr3	2.52 ± 0.51	2.00 ± 0.71	1.87 ± 0.40	1.43 ± 0.25	0.325
ccr9	3.24 ± 0.35	2.79 ± 0.16	2.93 ± 0.42	2.45 ± 0.24	0.329
ccr11	6.23 ± 1.09	7.70 ± 1.49	8.40 ± 1.48	7.40 ± 0.88	0.695
ck8 / ccl20	7.74 ± 0.62	5.90 ± 0.60	6.89 ± 0.50	6.10 ± 0.50	0.068
csf1r1	1.12 ± 0.08	1.18 ± 0.09	1.28 ± 0.08	1.14 ± 0.08	0.594
igm	5.09 ± 0.54	4.21 ± 0.67	5.40 ± 1.03	5.73 ± 0.59	0.221
igt-m	0.41 ± 0.08	0.24 ± 0.04	0.35 ± 0.04	0.23 ± 0.02	**0.039**
lgals1	11.38 ± 1.25	8.56 ± 0.52	9.60 ± 0.97	8.71 ± 0.89	0.240
lgals8	7.34 ± 1.59	5.56 ± 0.82	5.12 ± 0.72	6.11 ± 1.01	0.698
tlr2	3.23 ± 1.08	3.75 ± 1.62	2.83 ± 0.83	2.10 ± 1.03	0.737
tlr5	0.06 ± 0.01	0.06 ± 0.01	0.05 ± 0.01	0.06 ± 0.01	0.844
tlr9	0.09 ± 0.01	0.08 ± 0.01	0.10 ± 0.01	0.06 ± 0.01	**0.046**
cd209d	1.54 ± 0.55	1.38 ± 0.65	0.76 ± 0.27	0.37 ± 0.15	0.361
cd302	15.69 ± 2.22	13.96 ± 1.79	14.11 ± 1.54	11.63 ± 1.50	0.401
mrc1	3.25 ± 0.81	3.22 ± 1.26	2.54 ± 0.67	1.29 ± 0.27	0.290
fcl	8.61 [a] ± 3.22	13.40 [ab] ± 7.50	8.10 [a] ± 3.53	0.38 [b] ± 0.11	**0.019**

[1]*P* values result from one-way ANOVA. Different superscript letters in each row indicate significant differences among dietary treatments (Tukey post-hoc test *P* <0.05).

Table S3. Primers for qPCR amplification in seabream.

Gene	Symbol	GenBank	Primer sequence
Proliferating cell nuclear antigen	*pcna*	KF857335	F: CGT ATC TGC CGT GAC CTG T<br>R: AGA ACT TGA CTC CGT CCT TGG
Transcription factor HES-1-B	*hes1-b*	KF857344	F: GCC TGC CGA TAT GAT GGA A<br>R: GGA GTT GTG TTC ATG CTT GC
Krueppel-like factor 4	*klf4*	KF857346	F: ACA TCA CCG CAC GCA CAC<br>R: AAC CAC AGC CCT CCC AGT C
Claudin-12	*cldn12*	KF861992	F: CTC TCA GGG CTA CAC ATC TAC CTA TGC<br>R: ACA TTC GTG AGC GGC TGG AG
Claudin-15	*cldn15*	KF861993	F: CCG ATT GTG GAA GTA GTG GCT CTG GT<br>R: CAG CAT CAC CCA ACC GAC GAA CC
Cadherin-1	*cdh1*	KF861995	F: TGC TCC ATA CAG CGT CAC CTT ACA<br>R: CTC GTT CAT CCT AGC CGT CCA GTT
Cadherin-17	*cdh17*	KF861996	F: GAT GCC CGC AAC CCA GAG<br>R: CCG TTG ATT CAC TGC CGT AGA C
Tight junction protein ZO-1	*tjp1*	KF861994	F: AAG CAG TAT TAC GGT GAC TCA<br>R: TGC ATC CCT GGC TTG TAG
Desmoplakin	*dsp*	KF861999	F: GCA GAA GGA GCA CGA GAC CATC<br>R: GGG TGT TCT TGT CGC AGG TGA A
Gap junction Cx32.2 protein	*cx32.2*	KF862000	F: CGA GGT GTT CTA TCT GCT CTG TA<br>R: CTT GTG GGT GCG AGT CCT
Coxsackievirus and adenovirus receptor homolog	*cxadr*	KF861998	F: CAT CAG AGG ACT ACG AGA GG<br>R: CAT CTT GGC AGC ATT TGG T
Intestinal-type alkaline phosphatase	*alpi*	KF857309	F: CCG CTA TGA GTT GGA CCG TGA T<br>R: GCT TTC TCC ACC ATC TCA GTA AGG G
Liver type fatty acid-binding protein	*fabp1*	KF857311	F: GTC CTC GTC AAC ACC TTC ACC AT<br>R: CGC CTT CAT CTT CTC GCC AGT
Intestinal fatty acid-binding protein	*fabp2*	KF857310	F: CGA GCA CAT TCC GCA CCA AAG<br>R: CCC ACG CAC CCG AGA CTT C
Ileal fatty acid-binding protein	*fabp6*	KF857312	F: ACC CAG GAC GGC AAT ACC<br>R: CGA CGG TGA AGT TGT TGG T

Mucin 2	*muc2*	JQ277710	F: ACG CTT CAG CAA TCG CAC CAT<br>R: CCA CAA CCA CAC TCC TCC ACA T
Mucin 13	*muc13*	JQ277713	F: TTC AAA CCC GTG TGG TCC AG<br>R: GCA CAA GCA GAC ATA GTT CGG ATA T
Tumor necrosis factor-alpha	*tnf-alpha*	AJ413189	F: CAG GCG TCG TTC AGA GTC TC<br>R: CTG TGG CTG AGA GCT GTG AG
Interleukin-1 beta	*Il1b*	AJ419178	F: GCG ACC TAC CTG CCA CCT ACA CC<br>R: TCG TCC ACC GCC TCC AGA TGC
Interleukin-6	*il6*	EU244588	F: TCT TGA AGG TGG TGC TGG AAG TG<br>R: AAG GAC AAT CTG CTG GAA GTG AGG
Interleukin 7	*il7*	JX976618	F: CTA TCT CTG TCC CTG TCC TGT GA<br>R: TGC GGA TGG TTG CCT TGT AAT
Interleukin-8	*il8*	JX976619	F: CAG CAG AGT CTT CAT CGT CAC TAT TG<br>R: AGG CTC GCT TCA CTG ATG G
Interleukin-10	*il10*	JX976621	F: AAC ATC CTG GGC TTC TAT CTG<br>R: GTG TCC TCC GTC TCA TCT G
Interleukin 12 subunit beta	*il12b*	JX976624	F: ATT CCC TGT GTG GTG GCT GCT<br>R: GCT GGC ATC CTG GCA CTG AAT
Interleukin 15	*il15*	JX976625	F: GAG ACC AGC GAG CGA AAG GCA TCC<br>R: GCC AGA ACA GGT TAC AGG TTG ACA GGA A
Interleukin 34	*il34*	JX976629	F: TCT GTC TGC CTG CTG GTA G<br>R: ATG CTG GCT GGT GTC TGG
CD4	*cd4*	AM489485	F: TCCTCCTCCTCGTCCTCGTT<br>R: GGTGTCTCATCTTCCGCTGTCT
CD8 beta	*cd8b*	KX231275	F: CCGAAATGTGGAAGACTGGAACTC<br>R: CTTTGGAGGTAAGGTTGGAGGGAT
C-C chemokine receptor type 3	*ccr3*	KF857317	F: CTA CAT CAG CAT CAC CAT ACG CAT CCT<br>R: TGG CAC GGC ACT TCT CCTTCA
C-C chemokine receptor type 9	*ccr9*	KF857318	F: TCC CTG AGT TAA TCT TCG CCC AAG TG<br>R: TGT TGT ATT CGT TGT TCC AGT AGA CCA GAG
	ccr11	KF857319	F: GCT ACG ATT ACA GTT ATG AA

C-C chemokine receptor type 11			R: TAG ATG ATT GGG AGG AAG
C-C chemokine CK8 / C-C motif chemokine 20	*ck8/ cl20*	GU181393	F: CCG TCC TCA TCT GCT TCA TAC T<br>R: GCT CTG CCG TTG ATG GAA C
Macrophage colony-stimulating factor 1 receptor 1	*csf1r1*	AM050293	F: TTG CGT GTG GTG AGG AAG GAA GGT<br>R: AGC AGG CAG GGC AGC AGG TA
Immunoglobulin M	*igm*	JQ811851	F: ACC TCA GCG TCC TTC AGT GTT TAT GAT GCC<br>R: CAG CGT CGT CGT CAA CAA GCC AAG C
Immunoglobulin T membrane-bound form	*igt-m*	KX599201	F: AGA CGA TGC CAG TGA AGA GGA TGA GT<br>R: CGA AGG AGG AGG CTG TGG ACC A
Galectin-1	*lgals1*	KF862003	F: GTG TGA GGA GGT CCG TGA TG<br>R: ACT GTA GAG CCG TCC GAT AGG
Galectin-8	*lgals8*	KF862004	F: GGC GGT GAA CGG CGG TCA<br>R: GCT CCA GCT CCA GTC TGT GTT GAT AC
Toll like receptor 2	*tlr2*	KF857323	F: CAT CTG CGA CTC TCC TCT CTT CCT<br>R: ATT CAA CAA TGG AGC GGT GGA CTT
Toll like receptor 5	*tlr5*	KF857324	F: TCG CCA ATC TGA CGG ACC TGA G<br>R: CAG AAC GCC GAT GTG GTT GTA AGA C
Toll like receptor 9	*tlr9*	AY751797	F: GCC TTC CTT GTC TGC TCT TTC T<br>R: GCC GTA GAG GTG CTT CAG TAG
CD209 antigen-like protein D	*cd209d*	KF857327	F: CGC CAC GAG CAT GAG GAC AA<br>R: TCT TGC CAG AAT CCA TCA CCA TCC A
CD302 antigen	*cd302*	KF857328	F: GGA CCA GAG GAA GAG CAC ATC<br>R: GAC CAG GGC GGA CAT CAG
Macrophage mannose receptor 1	*mrc1*	KF857326	F: CTT CCG ACC GTA CCT GTA CCT ACT CA<br>R: CGA TTC CAG CCT TCC GCA CAC TTA
Fucolectin	*fcl*	KF857331	F: CCA TAC TGC TGA ACA GAC CAA CC<br>R: TGA TGG AGG TGA CGA TGT AGG A
